# Editorial: Expert opinions and perspectives in immunity to worms infection: 2022

**DOI:** 10.3389/fimmu.2023.1210743

**Published:** 2023-05-05

**Authors:** Luis Fonte, Boon Huat Lim, Maria E. Sarmiento, Armando Acosta

**Affiliations:** ^1^ Department of Parasitology, Institute of Tropical Medicine “Pedro Kourí”, Havana, Cuba; ^2^ School of Health Sciences, Universiti Sains Malaysia, Kubang Kerian, Kelantan, Malaysia

**Keywords:** helminth immunity, trichinellosis, thelaziasis, sparganosis, neurocysticercosis

Approximately 1,5 billion people worldwide, or nearly 20% of the world’s population, are infected with helminths (worms), a complex group of eukaryotic organisms that can survive for very long periods in the bloodstream, lymph, liver, or gastrointestinal tract of their hosts (1). Worm infections are a major public health problem, particularly for children living in low- and middle-income countries, due to their ability to cause significant morbidity (usually not fatal), stunted growth, impaired cognitive development, and socioeconomic loss (Weatherhead et al.) (1–2). Anthelmintic treatments, although highly successful in some circumstances, have the disadvantage of being relatively ineffective in the long term because of continued exposure to infective eggs and larvae in the immediate environment, the logistical complexities experienced in the distribution of drugs, and the emergence of drug resistance (2). On the other hand, vector control interventions, which could help in the control of some helminth infections, also face social, logistical, and economic difficulties (3). In this scenario, the study of immunity to helminth infections is necessary, both for a better understanding of their pathogenesis, the development of new diagnostic procedures, the delineation of successful host immune responses to develop protective vaccines against worms, and the understanding of parasite strategies involved in the establishment of chronic infection.

To control helminth infections, the host usually develops type 2 immune responses that promote important effector mechanisms, such as mucus secretion, which is crucial for helminth expulsion, and collagen deposition, which is critical for wound healing (4, 5). Nevertheless, the host-helminth interaction has at least two additional outcomes: (i) the classic and best-known downregulation of Th1 and Th17 type responses by Th2 mediators (4, 6), and (ii) helminth limitation of both host type 1 and type 2 responses by enhancing FOXP3+ T regulatory cells, B regulatory cells, and alternatively activated macrophages (M2), among others, which together cause the release of regulatory cytokines such as IL-10 and transforming growth factor (TGF-β) (Fonte et al.) (5, 7). The ability of helminths to modulate the immune responses of their respective hosts, in addition to allowing worms to survive in parasitized animals, also attenuates the inflammatory effects of host defensive mechanisms, thereby reducing the frequency and intensity of immunopathological events in them (Fonte et al.) (6, 8). Taking these basic considerations as a starting point, we briefly outline the contributions published in the Research Topic *Expert Opinions and Perspectives in Immunity to Worm Infection: 2022*.

It has been suggested that the production of mucus, which stimulates the expulsion of helminth intestinal forms, has evolved to protect against infection by these parasites (9). Jin et al. demonstrate that lentinan (LNT), a mushroom-extracted β-glucan, could induce *Trichinella spiralis* expulsion by promoting the defensive functions of the mucus layer through alteration of the gut microbiota. This finding, the authors say, suggests that LNT could be used in an easily implementable strategy to improve the host’s defenses against *T. spiralis* infection.

Macrophages can be differentiated into classically activated macrophages (M1) and alternatively activated macrophages (M2) under different conditions. In helminth infections, macrophages show predominant M2 activation or dynamic M1 to M2 polarization (10). Yin et al. show the role of *Thelazia callipaeda* macrophage migration inhibitory factor (T.cp-MIF) in inducing M1 to M2 type polarization of THP-1-derived macrophages. The understanding of the mechanisms of this polarization, which occurs *via* TLR4-mediated activation, could be useful for the development of treatments and vaccines against *T. callipaeda.*

Human sparganosis is caused by cestode larvae (spargana) of the genus *Spirometra* (11). The evaluation of the efficacy of anthelmintic treatment of this disease is mainly based on the remission of clinical symptoms and positive changes in cerebral lesions observed by magnetic resonance imaging (MRI) procedures, which are very expensive and not always available (12). The study by Xiang et al. provides evidence that the measurement of serum IgG antibody levels against sparganum is a promising tool for the evaluation of patients with cerebral sparganosis after treatment, circumventing the need to use MRI.

Neurocysticercosis (NCC), the infection of the human central nervous system with *Taenia solium* cysts, is the leading cause of acquired epilepsy worldwide (13). Subarachnoid NCC, which occurs when cysts of the parasite develop in the basal subarachnoid region or Sylvian fissure of the brain, is the less common but more severe form of extraparenchymal NCC (14). In an Opinion article on this special issue, Orrego et al. briefly review the molecular and cellular basis of subarachnoid NCC. In their work, the authors analyze the possible scenarios that would be interesting to address in order to better understand a disease that is still endemic in many parts of the world.

In another Opinion article on this Research Topic, Chin et al. suggest that in chronic helminth infection, parasite modulation of host immunity could downregulate the Th1 and Th17 immune responses against *Mycobacterium tuberculosis* (Mtb) infection and lead to progression from latent Mtb infection (LTBI) to active tuberculosis (ATB). Helminth immune modulation, the authors argue, could also cause indeterminate results in LTBI diagnostic tests and poor immunogenicity from BCG vaccination. In perspective, they remark that the immune environment of high TGF-β levels associated with helminth infections suggests the use of TB mucosal immunization as an alternative, or complement, to “classic” Th1-inducing vaccines in co-infected settings.

More than 1 billion people worldwide are infected with helminths, which trigger a wide range of immune responses against them and, at the same time, have potent effects on immunity of their hosts. Taken together, the small collection of works included in this Research Topic is a modest contribution to our understanding of the immunity to helminth infections, including both protective immune responses against worms and immune modulation by helminths. As guest Topic Editors, we hope that the Research Topic Expert Opinions and Perspectives in Immunity to Worm Infection: 2022 has provided readers with data and useful opinions on a complex and fascinating subject. Finally, we would like to thank all the authors, editors, and reviewers who contributed in different and valuable ways to the publication of those studies.

## Author contributions

All authors listed have made a substantial, direct, and intellectual contribution to the work, and approved it for publication.
